# Pediatric Burns in the Bedouin Population in Southern Israel

**DOI:** 10.1100/tsw.2007.239

**Published:** 2007-11-12

**Authors:** Arnon D. Cohen, R. Gurfinkel, R. Glezinger, Y. Kriger, N. Yancolevich, L. Rosenberg

**Affiliations:** ^1^ Clalit Health Services, Ben-Gurion University of the Negev, Beer-Sheva, Israel; ^2^ Siaal Research Center for Family Medicine and Primary Care, Ben-Gurion University of the Negev, Beer-Sheva, Israel; ^3^ Department of Plastic and Reconstructive Surgery, Center for R & D in Plastic Surgery, Soroka Medical Center, Beer-Sheva, Israel; ^4^ Ben-Gurion University of the Negev, Beer-Sheva, Israel

**Keywords:** Bedouin, burns, children, public health

## Abstract

Burn trauma is an important public health concern, with increased risk for burns in children. A cross-sectional study was performed to describe the epidemiological characteristics and risk factors for burns in hospitalized Bedouin children in Soroka University Medical Center during the years 2001–2002. In a population of 558 hospitalized burn-injured patients, 282 Bedouin children were identified. Two hundred and sixty five patients (94.0%) had burns involving less than 20% of the body surface area. Cause of the burns was scald in 190 patients (67.4%), fire in 80 patients (28.4%), chemical in 8 patients (2.8%), and explosion in 2 patients (0.7%). Two female patients (0.7%) aged 11 and 17 years died of their burns that were caused by fire. The mean length of hospitalization was 9.8 days. Pediatric burn injury has become a significant public health problem in the Bedouin population of the Negev. To reduce the burden of burn injury, it is necessary to increase current efforts in prevention of burns.

## INTRODUCTION

Burn injuries in childhood range from minor to severe events and present a major public health problem. Apart from causing death, burns often result in disastrous long-term effects that include disabling scars and contractures, and have substantial psychological consequences to the affected children. The younger the age at the time of the burn, the greater the loss in productive years. Risk factors for pediatric burns include low socio-economic status, poor living conditions, illiteracy, over-crowding, and floor-level cooking. Treatment of burns is expensive and consumes medical resources, as the care of a severe burn patient requires specialized staff and medical technologies[1-5].

The Bedouins in the Negev comprise about 130,000 people. They are an ethnic (Arab), religious (Islam) and cultural minority in Israel, and are in transition from a nomadic to a more settled and urban form of life. At present, this transition creates socio-economic problems including a high unemployment rate. Approximately half of the population lives in urban settlements; the other half lives in unrecognized rural settlements, a remnant of their nomadic life. In many cases, these settlements are self-proclaimed settlements and initiatives to take possession of public property. The public medical services offered by the health authorities are formally limited to established primary, secondary, and tertiary medical facilities that do not reach the loosely scattered, unrecognized settlements.

The transition from a traditional to a western lifestyle, characterized by changes in dietary habits and a reduction in physical activity, is associated with substantial changes in morbidity patterns such as a considerable increase in prevalence of diabetes[6] and home-related injuries[7].

Epidemiological studies on childhood injuries specific to the Bedouin ethnic group in Israel are scant due to difficulties in isolating specific ethnic groups in the general Israeli heterogeneous population[8]. Information on the exact magnitude of burn injuries in the different ethnic groups has not been well documented. The hazards of household-related burns are known to occur in families where many children live in small quarters, where hot liquids are an important part of the food habitudes, when open fires are used, when cooking and heating means are badly and offhandedly organized, and when much of the daily life is done on the floor. All these conditions exist in the Bedouin community. It is not surprising that many of these children fall victim to the dreaded burn injury. In order to be able to design and implement an effective prevention effort, it is essential to define the epidemiology and causes that should be addressed to achieve the maximum benefit.

In this study, we describe 282 severely burned Bedouin children who were hospitalized in the years 2001 and 2002 in Soroka University Medical Center.

## MATERIALS AND METHODS

During the years 2001–2002, 282 burn-injured children were hospitalized in the Burns and Plastic Surgery Department in Soroka University Medical Center, Beer-Sheva, Israel. All the pediatric burns were analyzed retrospectively in terms of severity, extent, causes, complications, risk factors, and overall mortality.

Statistical analysis was performed using SPSS software. Results of continuous variables are shown as means ± SD. Results of categorical variables are described as frequencies. Chi-square tests were used to statistically analyze significant differences of categorical variables. *T*-tests were used to compare continuous parameters between the groups. P values ≤.05 were considered statistically significant.

## RESULTS

Two hundred and eighty-two burn-injured children were hospitalized during the years 2001–2002. There were 220 children younger than 4 years-of-age (78.0%), 43 children between 4–10 years (15.3%), and 19 children (6.7%) above 10 years. The age distribution of Bedouin children hospitalized for burns is shown in Table 1. There were 154 male patients (54.6%). Burns occurred more frequently in males compared to females, particularly in older children (Table 1).

Ninety four percent of the children suffered from burn injuries of less than 20% of body Surface area (BSA). Older children were hospitalized for more extensive burns, compared to younger children (p value<0.001; Table 2). The majority of the burns were second degree; the depth of the burns is depicted in Table 3. The patients were hospitalized for a mean period in of 9.8 days (Table 4). Scald was the most common cause of burn injury (67.4%) followed by flame and chemical burns. Electrical burns were not observed in this patient sample (Table 5).

Eight patients (2.8%) had smoke inhalation in addition to the burns. Twenty-eight patients (9.9%) were admitted to the intensive care unit. The overall mortality was 0.7%; the two victims were females aged 11 and 17 years, suffering from a burn over 90% and 97% of the total BSA. Flame was the cause of the burn in both patients. Both patients suffered from smoke inhalation injury. Septic shock and multi-organ failure were the cause of death.

**Table 1.**
**Age and Gender Distribution of Bedouin Children Hospitalized for Burns**

| Age | | Gender of patients | | Total |
|---|---|---|---|---|
| | | Male | Female | |
| <4 years | Frequency | 116 | 104 | 220 |
| | Percent | 52.7 | 47.3 | 100.0 |
| 4–10 years | Frequency | 25 | 18 | 43 |
| | Percent | 58.1 | 41.9 | 100.0 |
| >10 years | Frequency | 13 | 6 | 19 |
| | Percent | 68.4 | 31.6 | 100.0 |
| Total | Frequency | 154 | 128 | 282 |
| | Percent | 54.6 | 45.4 | 100.0 |

**Table 2.**
**Extent of the Burn, Stratified According to Patient Age**

| BSA | Age (years) | | | Number of patients |
|---|---|---|---|---|
| | <4 years | 4–10 years | >14 years | |
| 0–20 | 210 | 40 | 15 | 265 |
| | 95.5 | 93.0 | 78.9 | 94.0 |
| 20–40 | 9 | 3 | 1 | 13 |
| | 4.1 | 7.0 | 5.3 | 4.6 |

**Table 3.**
**Depth of the Burns.**

| | Frequency | Percent |
|---|---|---|
| First degree | 4 | 1.4 |
| Second degree (shallow) | 225 | 79.8 |
| Second degree (deep) | 36 | 12.8 |
| Third degree | 17 | 6.0 |
| Total | 282 | 100.0 |

**Table 4.**

**Length of Hospitalization According to %BSA Involved in the Burn**

| %BSA | n | Mean | SD | Minimum | Maximum |
|---|---|---|---|---|---|
| 0–20 | 166 | 8.0 | 7.9 | 1 | 45 |
| 20–40 | 6 | 29.8 | 14.4 | 9 | 51 |
| 40–60 | 2 | 85.5 | 53.0 | 48 | 123 |
| 60–100 | 2 | 18.5 | 13.4 | 9 | 28 |
| Total | 176 | 9.8 | 12.8 | 1 | 123 |

**Table 5.**
**Cause of the Burn**

| | Frequency | Percent |
|---|---|---|
| Scald | 190 | 67.4 |
| Flame | 80 | 28.4 |
| Chemical | 8 | 2.8 |
| Explosion | 2 | 0.7 |
| Other | 2 | 0.7 |
| Total | 282 | 100.0 |

## DISCUSSION

Burn injury in children is a significant public health concern. Severe burns may be lethal and survivors often suffer from disfiguring and disabling scar, psychological trauma and loss of productive years for affected patients. Almost every child who has sustained a burn injury in childhood will have some sequel of the accident persisting into adulthood[9-12]. The care of burn patients is complicated and expensive, and consumes considerable health care system and personal resources. A national program for burn and house-related accident prevention was instituted in Israel 15 years ago. This program has contributed to increased awareness of risks by children and to enhanced injury control[13,14].

Previous studies conducted in Israel illustrated the epidemiology of burn injury in children[8,15-19]. In the current study, we described 282 burned Bedouin children who needed to be hospitalized during the years 2001–2002 in Soroka University Medical Center in Beer-Sheva, Israel. The profile of pediatric burn patients in the Bedouin population corresponds with characteristics of reported studies in both developed and developing countries[2,4,5,20].

In the current study, 78% of the patients were <4 years-of-age, and 22% were between 4 and 18 years-of-age. The age distribution is similar to observations by Silfen et al.[21] who described 282 pediatric burn patients who were treated in the emergency department of Schneider Children's Medical Center in 1996. Schneider Children's Medical Center is located in Tel-Aviv, a large metropolitan area where Bedouin are not represented as a well-defined community. Our observations are also similar to observations made by Kumar et al.[20] who described 309 burn injuries in children who were treated between 1989–1998 in Kasturba Hospital, Manipal (India). Kumar et al.[20] observed that 76.1% of the patients were <5 years of age, whereas 23.9% of patients were between 6 and 10 years-of-age.

In our study, there were 54.6% male patients and 45.4% female patients. These observations are similar to those described by Silfen et al.[21], who observed that 62.8% of the patients were male; however, Kumar et al.[20] found that females were affected more than males (74.1 vs. 25.9%).

The overall mortality of hospitalized Bedouin children in the current study was 0.7%. The two victims were females, 11 and 17 years-of-age who suffered burns in 90% and 97% of the total BSA. In both patients, burns were caused by flame and were associated with smoke inhalation. These observations are similar to observations by Silfen et al.[21], who described a 1% mortality rate in their series. However, Davies[1], who surveyed almost 3500 hospital admissions and published reports over the past 30 years, observed a mortality rate from burns in India between 6 and 20% for children (0–10 years old). In the study by Kumar et al.[20], overall pediatric mortality was 7.4%, mortality was decreased in children <5 years-of-age compared to children older than 5 years-of-age (6.3 vs. 10.8%).

Scald was the most common cause of burn injuries (67.4%) in our study. This observation is similar to other studies[20,21] and reflects overcrowding in Bedouin families, where young children play in the vicinity of the cooking area, leading to scalds from spilled hot fluids or foods. In our study, the second most common cause for burns was flame (28.4%), a figure similar to the study in India by Kumar et al.[20] who observed that flames caused 22.7% of the burns, and are in contrast with the low rate of flame burns observed by Silfen et al.[21], who observed that only 5.1% of burns were caused by flames. The high rate of flame injury observed in our study reflects the exposure to open fire in Bedouin families, which is an infrequent risk in central Israel, as observed by Silfen et al.[21] Since the kitchen or cooking area and open fires are the most dangerous for pediatric patients, certain steps could and should be taken to intervene in these specific areas, preventing the possibility of burn injuries in Bedouin children.

The development of the burn center and its comprehensive approach and services in Soroka University Medical Center provides considerable benefit. A distinct advantage for patients in Soroka University Medical Center is the availability of multi-disciplinary care. A psychiatrist, dietitian, social worker, neurologist, pediatrician, anesthesiologist, neurosurgeon, ophthalmologist, physician, physiotherapist, and occupational therapist, as well as a comprehensive and long-established prevention campaign are all available under one roof. An in house skin bank, bilayered skin substitutes, and keratinocyte culture are immediately available free of charge at the Medical Center.

This study pinpoints our attention to a specific group at risk for burn injury in a well-defined community. Some of the risk factors such as cooking and heating techniques, appliances and behavior trends should be changed. Other socio-economic and cultural factors are more difficult to handle. Issues such as family size and composition, level of education are more difficult. Many of these factors are deeply rooted in religious and cultural concepts (e.g., number of wives or number of children) with secondary gains that complicate the situation even more. Fortunately, our duty is very simple: to treat the injured and prevent future injuries as much as we can. We do not have the authority or the means to change religious and social attitudes. Thus, we will continue to develop and implement our prevention project and treat the injured children.

In conclusion, pediatric burn injury has become a significant public health problem among the Bedouin population of the Negev. To reduce the burden of burn injury, it is necessary to increase current efforts in patient education. The southern district of Clalit Health Services currently performs a focused intervention campaign for the control of pediatric burns and injury in the Bedouin population. New initiatives may also be required, such as aggressive campaigns to increase the awareness of burn injury in the Bedouin population. We recommend that sufficient resources be allocated for burn injury control in the Bedouin population.
